# miR-564 acts as a dual inhibitor of PI3K and MAPK signaling networks and inhibits proliferation and invasion in breast cancer

**DOI:** 10.1038/srep32541

**Published:** 2016-09-07

**Authors:** Merve Mutlu, Özge Saatci, Suhail A. Ansari, Emre Yurdusev, Huma Shehwana, Özlen Konu, Umar Raza, Özgür Şahin

**Affiliations:** 1 Department of Molecular Biology and Genetics, Faculty of Science, Bilkent University, 06800 Ankara, Turkey

## Abstract

Dysregulation of PI3K and MAPK pathways promotes uncontrolled cell proliferation, apoptotic inhibition and metastasis. Individual targeting of these pathways using kinase inhibitors has largely been insufficient due to the existence of cross-talks between these parallel cascades. MicroRNAs are small non-coding RNAs targeting several genes simultaneously and controlling cancer-related processes. To identify miRNAs repressing both PI3K and MAPK pathways in breast cancer, we re-analyzed our previous miRNA mimic screen data with reverse phase protein array (RPPA) output, and identified miR-564 inhibiting both PI3K and MAPK pathways causing markedly decreased cell proliferation through G1 arrest. Moreover, ectopic expression of miR-564 blocks epithelial-mesenchymal transition (EMT) and reduces migration and invasion of aggressive breast cancer cells. Mechanistically, miR-564 directly targets a network of genes comprising AKT2, GNA12, GYS1 and SRF, thereby facilitating simultaneous repression of PI3K and MAPK pathways. Notably, combinatorial knockdown of these target genes using a cocktail of siRNAs mimics the phenotypes exerted upon miR-564 expression. Importantly, high miR-564 expression or low expression of target genes in combination is significantly correlated with better distant relapse-free survival of patients. Overall, miR-564 is a potential dual inhibitor of PI3K and MAPK pathways, and may be an attractive target and prognostic marker for breast cancer.

Breast cancer is one of the major common malignancies among women worldwide. Molecular heterogeneity accompanied by alterations in signaling pathways are the major factors that collectively lead to enhanced cellular growth, differentiation, reduced apoptosis and development of drug resistance in breast cancer^1^. Phosphatidylinositol 3-kinase (PI3K) and the mitogen activated protein kinase (MAPK) cascades are among the most common dysregulated signaling pathways in breast cancer. PI3K pathway is the most activated oncogenic pathway in 70% of tumors in patients with invasive breast cancer^2^. Mechanisms leading to hyperactivation of PI3K pathway include PIK3CA mutation (~30%), PIK3CA copy number gain, loss of PTEN protein and AKT activation^3^. AKT stimulation triggers a series of downstream effects, such as activation of transcription factors p53 and NF-κB, which have major impact on cancer cell growth and survival^4^,^5^. Aberrantly activated MAPK pathway, on the other hand, has been observed in 2–10% of breast cancer patients^3^,^6^,^7^,^8^. ERK1/2 activation by phosphorylation is an important marker for stimulated MAPK pathway and shown to be elevated in 50% of primary breast tumors in comparison to adjacent normal tissue^9^. Activated ERK phosphorylates series of cytoplasmic and nuclear target proteins. ERK nuclear targets including TCF (Ternary Complex Factor) play key roles in activation of c-myc, c-jun and CREB transcription factors, which are important regulators of tumorigenesis^10^.

Over the years, several efforts have been made to inhibit cancer progression using small molecule inhibitors targeting specific kinases in the PI3K or MAPK pathways^11^,^12^. However, such approaches often results in limited success either at experimental phase or in clinical trials, mostly due to mutual cross-talk of PI3K and MAPK pathways interfering with the efficacies of these inhibitors^11^. For example, it has been shown that MAPK pathway is activated in the presence of RAD001, an mTOR inhibitor, by activating S6K-PI3K-Ras feedback loop^13^. Another study demonstrates that ERK suppression by MEK inhibitor increases EGF-stimulated AKT activation^14^,^15^. On the other hand, combinatorial inhibition of both pathways often raises the high toxicity concerns^16^,^17^. In this regard, several studies have stipulated selecting the patients with genomic features more suitable for clinical response to combined inhibition of PI3K and MAPK pathways. For example, such dual-targeting strategy is shown to be mainly effective in patients with concurrent PI3K pathway genetic alteration and *KRAS* or *BRAF* mutation, thus necessitates molecular genetic profiling of patients before inhibitors treatment^17^,^18^,^19^. Our previous data also suggest that treatment with PI3K/mTOR inhibitor BEZ235 results in ERK upregulation, while subsequent treatment with lapatinib in combination with BEZ235 results in weight loss and decreased mobility of transgenic mouse models despite significant reduction in tumor volume^20^,^21^. These studies suggest that more research is needed to identify novel strategies to target these pathways simultaneously in a more effective manner.

MicroRNAs are short (18–25 nucleotides), non-coding RNAs, which bind mostly to the 3′-untranslated region (3′-UTR) of target mRNAs resulting in either mRNA cleavage or translational repression^22^. Human genome codes for more than 2600 miRNAs (miRBase Release 21), and it has been predicted that 60% of the genome is regulated by miRNAs^23^. Role of miRNAs in cancer regulation was first established 14 years ago and over the years, growing number of evidences have clearly indicated the involvement of dysregulated miRNAs in cancer cell proliferation, invasion, metastasis and drug resistance^24^,^25^. MicroRNAs can be classified either as tumor suppressor or oncogenic depending on their tissue-specific expression patterns and the targets they regulate. In other words, miRNAs negatively regulating oncogenes may function as tumor suppressors while those targeting tumor suppressor genes serve as oncogenic miRNAs (oncomiRs)^26^,^27^. Furthermore, miRNAs tend to regulate several biological processes by not targeting a single gene, but combination of many genes in a network-based manner. In this regard, several studies including ours demonstrate the interactions between microRNAs and many genes as their targets in human cancer^28^,^29^. Therefore, recent work focuses on testing the potential of using miRNAs as drugs or drug targets. Importantly, miR-122 inhibitor (miravirsen) has been tested in Phase 2a trial in chronic hepatitis C patients (NCT01200420)^30^ while miR-34 (MRX34) has been tested in Phase 1 trial in liver cancer patients (NCT01829971A)^31^. Thus, miRNAs have a high potential as targeted therapy for cancer treatment by modulating several targets simultaneously.

In this study, we aimed at identifying a microRNA-based approach to target both PI3K and MAPK pathways simultaneously to inhibit cell proliferation and invasion in breast cancer cells. Our findings suggest that miR-564 is a potential tumor suppressor that regulates both PI3K and MAPK signaling by directly targeting a network of genes (AKT2, GNA12, GYS1 and SRF) linked to these pathways, and thus controls breast cancer cells proliferation, EMT, migration and invasion. Importantly, lower levels of miR-564 expression or higher levels of target genes expression is associated with poor prognosis and survival suggesting miR-564 or its targets in combination as a potential marker for breast cancer progression.

## Results

### miR-564 inhibits both PI3K and MAPK pathways and reduces the viability of breast cancer cells *via* a G1 cell cycle arrest

**Figure 1:**
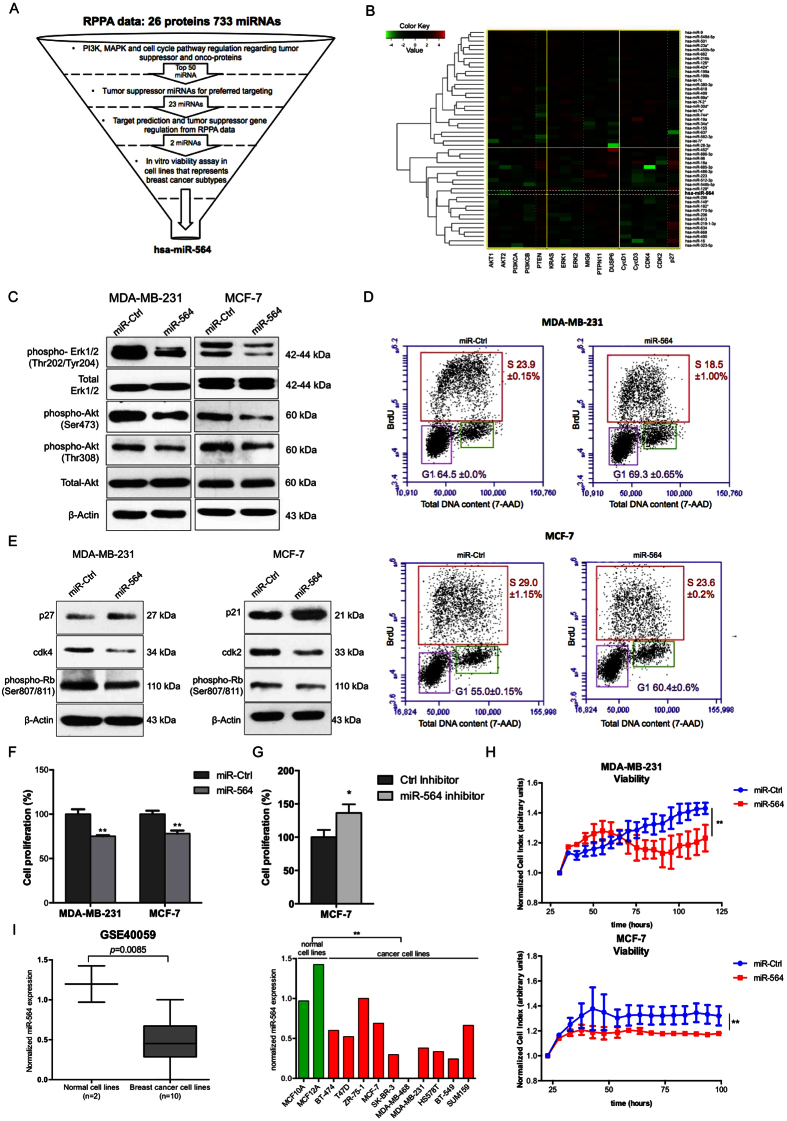
miR-564 suppresses both PI3K and MAPK pathways and reduces the viability of breast cancer cells *via* inducing a G1 cell cycle arrest. (**A**) Flowchart showing selection process for candidate miRNA using the data from miRNA mimic screen with RPPA output in MDA-MB-231 breast cancer cell line. (**B**) Heatmap depicting correlations of top 50 miRNAs with tumor suppressor or oncogenes from PI3K and MAPK pathways as well as cell cycle pathway from RPPA data. X-axis is classified into groups according to components involved in different pathways. Y-axis is clustered using hcluster from R conductor. The miRNAs above white line are potential oncogenes while those below are potential tumor suppressors. (**C**) Western blots showing decreased expression and phosphorylation of proteins linked with PI3K and MAPK pathways. Full length western blot images for phospho-Akt (T308) and phospho-ERK1/2 are shown in Supplementary Fig. 6. (**D**) BrdU/7-AAD staining of MDA-MB-231 and MCF-7 cell lines transiently transfected with miR-564 or scramble control. (**E**) Western blot analyses of G1/S transition proteins in miR-564 mimic transfected MDA-MB-231 and MCF-7 cells. (**F**) End-point cell viability assay performed upon miR-564 mimic transfection in MDA-MB-231 and MCF-7 cells. (**G**) End-point cell viability assay performed upon miR-564 hairpin inhibitor transfection in MCF-7 cells. (**H**) Real-time cell proliferation analysis in the presence of miR-564 transfection or control miRNA. Statistical significance of results was tested using paired two-tailed student t-test. (**I**) Expression of miR-564 in GSE40059 dataset with 12 different breast cell lines classified as cancerous versus normal (left panel). Expression in each cell line is depicted as relative expression with respect to MDA-MB-468 (right panel).

To find a potential dual inhibitor miRNA simultaneously targeting both PI3K and MAPK pathways, we re-analyzed our previously published reverse phase protein array (RPPA) data^28^ showing changes in the expression of 16 proteins involved in these two pathways and cell cycle progression upon transfection of MDA-MB-231 cell line with miRNA mimic library including 733 miRNAs (Fig. 1A). We clustered miRNAs according to the degree (proximity) of PI3K and MAPK pathways as well as cell cycle modulation upon their individual overexpression (Fig. 1B) (details are given in Methods section), and identified 23 potential tumor suppressor miRNAs negatively co-regulating PI3K, MAPK pathways and cell cycle progression (Supplementary Table 1). Among these 23 miRNAs, hsa-miR-564 and hsa-miR-490 were relatively less studied in the context of tumor formation and progression in breast cancer. In order to test their potential tumor suppressor role, we performed a viability assay on breast cancer cell lines representing different clinical subtypes (TNBC, ER+ and HER2-overexpressed) where miR-564 and miR-490 were ectopically expressed. We observed a superior growth inhibitory effect of miR-564 over miR-490, and thus decided to focus on miR-564 for further study (Supplementary Fig. 1A). For further experiments, miR-564 expression levels were verified in miR-564 transiently transfected MDA-MB-231, MCF-7 and MDA-MB-436 cell lines as well as in MDA-MB-231 cells stably transfected with miR-564 (Supplementary Fig. 1B).

To validate the RPPA data showing potential PI3K and MAPK pathway inhibition by miR-564, we examined AKT and ERK1/2 phosphorylation levels in miR-564 transfected MDA-MB-231 and MCF-7 cells, which represent triple negative breast cancer (TNBC) and estrogen receptor positive (ER+) breast cancer subtypes, respectively. A prominent decrease in the phosphorylation of both ERK1/2 and AKT was observed, whereas levels of total ERK1/2 and AKT remained unchanged upon miR-564 expression (Fig. 1C). Furthermore, we had observed a reduction in Cyclin D1, CDK2 and CDK4 accompanied by an increase in p27 level upon miR-564 expression in MDA-MB-231 cell line in RPPA experiment (Fig. 1B). This instigated us to test the effect of miR-564 on G1/S transition during cell cycle progression. We observed a G1 cell cycle arrest in MDA-MB-231 and MCF-7 cells transfected with miR-564 by both BrdU/7-AAD staining (Fig. 1D) as well as Western blot analysis of G1 and S phase associated cyclin dependent kinases (CDKs), CDK inhibitors and Retinoblastoma protein (Rb) phosphorylation, a key G1/S transition marker. The G1 arrest was found to be mediated by upregulation of p27 and downregulation of CDK4 in MDA-MB-231 cells whereas it has been regulated by upregulation of p21 and downregulation of CDK2 in MCF-7 cells (Fig. 1E). To test if the G1 arrest induced by miR-564 overexpression is also reflected to growth inhibition, in addition to an end-point viability assay, we performed Real-Time Cell Analyzer (RTCA) assay in MDA-MB-231 and MCF-7 cells and observed a reduction in cell viability in both cell lines (Fig. 1F,H). Subsequently, MCF-7 cells transfected with miR-564 antagonist showed increase in cell proliferation (Fig. 1G).

**Figure 2:**
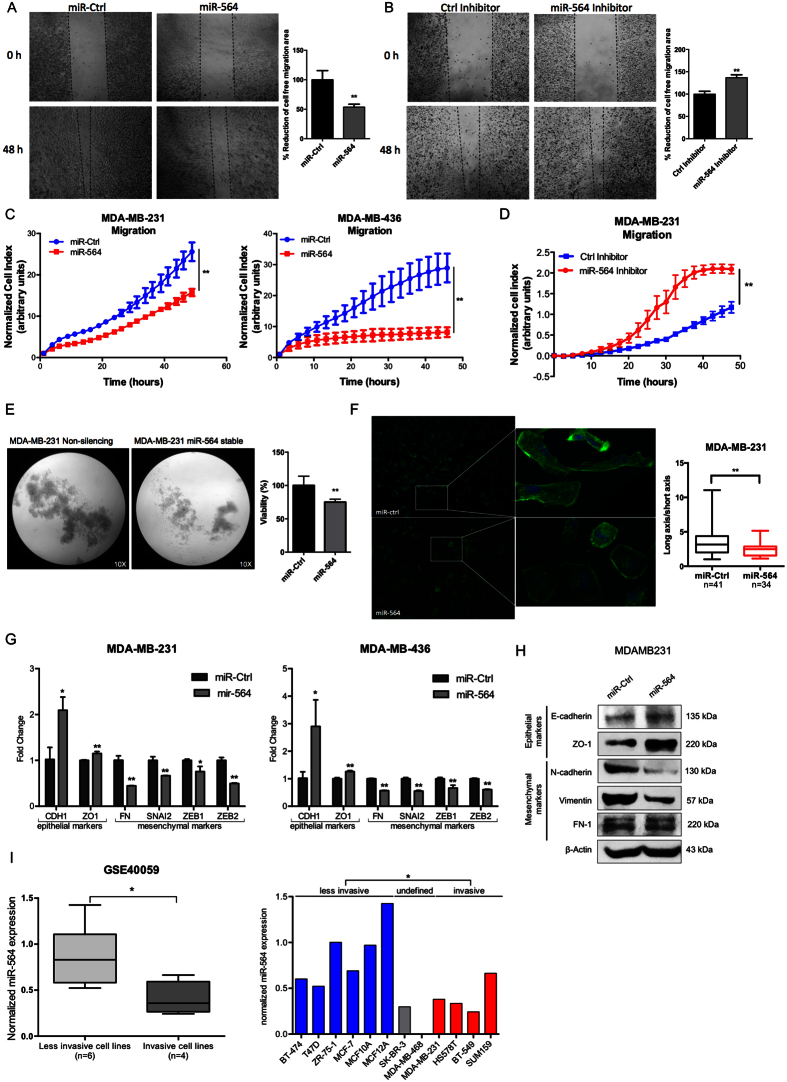
miR-564 inhibits migration and invasion of breast cancer cells by blocking EMT. (**A**) *In vitro* scratch assay with MDA-MB-231 cell line at 0 and 48 hours post-transfection with miR-564 or control miRNA. (**B**) Wound healing assay in MDA-MB-231 cell line at 0 and 48 hours post-transfection with miR-564 inhibitor or control hairpin inhibitor. Quantification was performed by the measurement of gap distance after cell migration. (**C**) Real-time migration assay using RTCA with MDA-MB-231 and MDA-MB-436 cells in the presence of miR-564 or control miRNA. (**D**) Real-time migration assay using RTCA with MDA-MB-231 cells upon miR-564 antagonist. (**E**) Poly-HEMA assay to analyze anchorage-independent growth of MDA-MB-231-luc cells stably-transfected with miR-564 and incubated for 48 hours. Control group was represented by cells stably-transfected with empty vector. Tumorigenicity was quantified as percent viable cells measured with WST-1 cell proliferation reagent. (**F**) DAPI/Phalloidin staining of MDA-MB-231 cell line. Images were taken 24 hours after transient transfection with miR-564 mimic. Cell morphology changes were quantified as ratio of long axis to short axis for each cell. (**G**) qRT-PCR showing the expression of epithelial and mesenchymal markers in MDA-MB-231 and MDA-MB-436 cell lines upon miR-564 mimic transfection. (**H**) Western blot showing changes in protein levels of EMT markers in miR-564 transfected MDA-MB-231 cells. (**I**) Expression of miR-564 in invasive and less-invasive breast cancer cell lines from GSE40059 dataset (left panel). Expression in each cell line is depicted as relative expression with respect to MDA-MB-468 (right panel).

To further support our *in vitro* findings, we analyzed several publicly available cell line datasets deposited in Gene Expression Omnibus (GEO) database. In GSE40059^32^ dataset, representing mRNA and miRNA expression profiles of a panel of 10 different breast cancer cell lines and 2 immortalized normal breast cell lines, we found that miR-564 expression level was lower in breast cancer cell lines as compared to normal breast cells (Fig. 2I).

Overall, our data show that miR-564 suppresses both PI3K and MAPK pathways, and reduces the growth capacity of breast cancer cell lines by inducing a G1 cell cycle arrest.

### miR-564 inhibits migration and invasion *via* blocking EMT in breast cancer cell lines

PI3K and MAPK pathways are known to induce epithelial-mesenchymal transition (EMT) and lead to migration and invasion in different cancers^33^. To test if miR-564 also affects metastasis-related processes by inhibiting these pathways, we first examined the effect of ectopic miR-564 expression on migration of breast cancer cell lines in wound healing and real-time migration assays (Fig. 2A,C). For these experiments, MDA-MB-231 and MDA-MB-436 cell lines were used as two different highly invasive breast cancer models. We observed a significant inhibition of migration in both MDA-MB-231 and MDA-MB-436 breast cancer cells with RTCA migration assay. In addition, migration capacity of MDA-MB-231 cells was significantly increased in the presence of miR-564 inhibitor transfection (Fig. 2B,D). miR-564 also significantly reduced anchorage-independent growth of MDA-MB-231 cells stably overexpressing miR-564 as assessed by poly-HEMA assay (Fig. 2E). Furthermore, we observed a more epithelial-like morphology when we overexpressed miR-564 in MDA-MB-231 cells as characterized by the loss of stress fibers, an increase in the expression of epithelial markers (CDH1 and ZO-1) and a decrease in expression of mesenchymal markers (FN, SNAI2, ZEB1 and ZEB2) at both RNA and protein levels (Fig. 2F–H). By using the same dataset showing lower levels of miR-564 in breast cancer cell lines as compared to normal cell lines (GSE40059), we compared expression of miR-564 between less invasive and invasive cells, and observed significantly lower levels of miR-564 in invasive breast cell lines (Fig. 2I). Overall, in line with the known roles of PI3K and MAPK pathways on metastasis-related processes, miR-564 acts as a dual inhibitor of these pathways and leads to inhibition of anchorage-independent growth, EMT, migration and invasion in breast cancer cells.

### miR-564 inhibits PI3K and MAPK pathways by targeting a network of genes in these pathways

**Figure 3:**
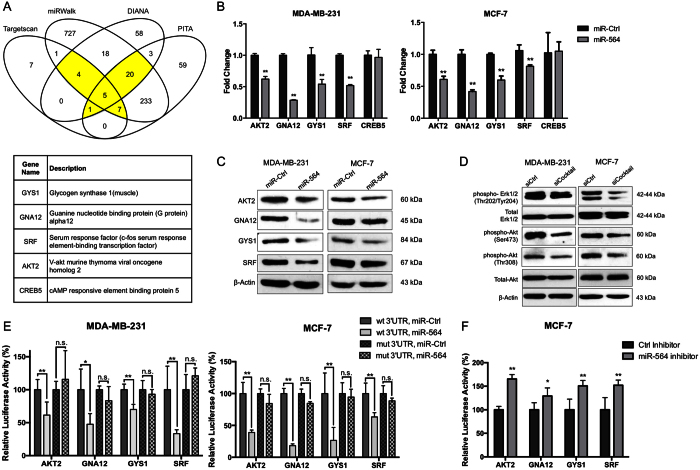
AKT2, GNA12, GYS1 and SRF are direct targets of miR-564 and involved in dual inhibition of PI3K and MAPK pathway. (**A**) Venn diagram showing potential targets of miR-564 from 4 different target prediction databases. Descriptions of selected genes are given in the table below. (**B**) qRT-PCR results of the expression of potential targets upon miR-564 transfection. (**C**) Western blot analysis of target proteins in miR-564 transfected MDA-MB-231 and MCF-7 cell lines. (**D**) Expression and phosphorylation levels of PI3K and MAPK pathway proteins in MDA-MB-231 and MCF-7 cells transfected with siCtrl or siCocktail. Full length western blot images for phospho-Akt (T308) and phospho-ERK1/2 are shown in Supplementary Fig. 6. (**E**) 3′-UTR luciferase reporter assay in MDA-MB-231 and MCF-7 cells by dual transfection with either mimic control or miR-564 along with psiCHECK2 vector harboring either wild type or mutated 3′-UTR sequence of each target gene. (**F**) 3′-UTR luciferase reporter assay in MCF-7 cells after individual transfection with psiCHECK2 vector DNA containing 3′-UTR of each target gene followed by treatment with anti-miR-564 or control hairpin inhibitor. Relative luciferase activity was measured as the ratio of *renilla/firefly* luciferase.

Having identified miR-564 as a regulator of cell viability, migration and invasion in breast cancer, we aimed at identifying the targets of miR-564 mediating these observed effects. To do this, we identified miR-564 predicted targets by using four different publicly available miRNA target prediction databases (Targetscan (Release 6.0)^34^, miRwalk (2.0)^35^, DIANA^36^ and PITA^37^). Predicted targets common to two or more databases were selected for further analysis (Fig. 3A), and genes found in PI3K and MAPK pathways among these 37 predicted targets were identified by performing KEGG pathway enrichment analysis^38^. Five genes, namely; V-akt murine thymoma viral oncogene homolog 2 (AKT2), guanine nucleotide binding protein subunit alpha 12 (GNA12), glycogen synthase 1 (GYS1), serum response factor (SRF) and cAMP responsive element binding protein 5 (CREB5) were among the predicted miR-564 targets playing role in either PI3K or MAPK pathways (Supplementary Fig. 2A). To experimentally validate targeting of these genes by miR-564, we examined the changes in their expression in MDA-MB-231 and MCF-7 cells upon miR-564 overexpression. Among the five predicted target genes, four of them (AKT2, GNA12, GYS1 and SRF) showed decrease at mRNA (Fig. 3B) and protein levels (Fig. 3C) in both cell lines; therefore selected for further analysis.

As miRNAs can function by targeting several genes in a network, an approach of using a cocktail of siRNAs against miR-564 targets (referred here as siCocktail) would better recapitulate the effects of miR-564 overexpression by mimic transfection and could be superior against silencing only one predicted target. Therefore, we used siCocktail to knockdown all four targets of miR-564 simultaneously. We have confirmed the silencing of AKT2, GNA12, GYS1 and SRF by siCocktail at mRNA (Supplementary Fig. 2B) and protein levels (Supplementary Fig. 2C). Decreased levels of ERK1/2 and AKT phosphorylation in both MDA-MB-231 and MCF-7 cell lines upon transfection with miR-564 (Fig. 1C) or siCocktail (Fig. 3D) in our Western blot analysis clearly indicated that the dual pathway inhibitory effects of miR-564 was, at least in part, *via* simultaneous targeting of AKT2, GNA12, GYS1 and SRF.

To validate the direct targeting of AKT2, GNA12, GYS1 and SRF by miR-564 as shown at mRNA and protein levels (Fig. 3B,C), we performed dual luciferase reporter assays after cloning the 3′-UTR of each individual target gene at the downstream of luciferase open reading frame (ORF). Co-transfection of 3′-UTR vectors with miR-564 mimic led to a reduction in luciferase activity in both MDA-MB-231 and MCF-7 cells compared to the miR-Control transfected cells. Subsequently, co-transfection of miR-564 with mutated miR-564 seed-matching sequence containing 3′-UTR vectors showed no significant change in luciferase activity (Fig. 3E). Conversely, in case of co-transfection of 3′-UTR vectors with a hairpin inhibitor against miR-564, we observed a significant increase in the luciferase activity in MCF-7 cell line (Fig. 3F). In conclusion, these data demonstrate that AKT2, GNA12, GYS1 and SRF are the direct targets of miR-564, and miR-564 inhibits PI3K and MAPK pathways *via* targeting a network of these genes.

### Combinatorial inhibition of AKT2, GNA12, GYS1 and SRF mimics the effects of miR-564 overexpression on cell proliferation, EMT, migration and invasion

**Figure 4:**
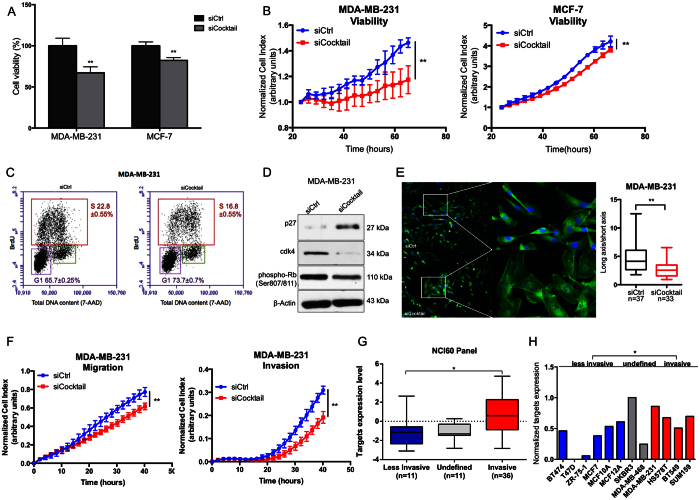
Combinatorial knockdown of AKT2, GNA12, GYS1 and SRF mimics the inhibitory effects of miR-564 on proliferation and migration in breast cancer cells. (**A**) End-point viability assay in MDA-MB-231 and MCF-7 cell lines 48 hours post-transfection with siCtrl or siCocktail. (**B**) Real-time cell proliferation results in MDA-MB-231 and MCF-7 cell lines transfected with siCtrl or siCocktail and incubated for 48 hours. (**C**) Cell cycle analysis of MDA-MB-231 cells after transfection with siCtrl or siCocktail using BrdU/7-AAD staining. (**D**) Western blot analysis of G1/S transition proteins in MDA-MB-231 cells transiently transfected with siCtrl or siCocktail. (**E**) DAPI/Phalloidin staining of MDA-MB-231 cells. Images were taken and quantified 24 hours after transient transfection with siCtrl or siCocktail. Cell morphological changes were quantified as a ratio of long axis to short axis for each cell. (**F**) Real-time migration and invasion analysis of MDA-MB-231 cells transfected with siCtrl or siCocktail using RTCA. (**G**) Expression levels of target genes in combination in less-invasive and invasive cancer cell lines from NCI60 panel. (**H**) Expression of target genes in combination in invasive and less-invasive breast cancer cell lines panel from GSE40059 dataset. Different probes of each gene were averaged.

To validate that AKT2, GNA12, GYS1 and SRF are the major functional targets of miR-564, we examined the effects of combinatorial inhibition of miR-564 targets using an siCocktail on various cellular processes including viability, migration and invasion. Both end-point and RTCA cell viability assays showed that siCocktail mimics the growth inhibitory effects of miR-564 in both MDA-MB-231 and MCF-7 cell lines (Fig. 4A,B). In addition, we observed that combinatorial inhibition of miR-564 target genes using siCocktail led to a G1 cell cycle arrest in MDA-MB-231 cells similar to the effect seen with miR-564 overexpression which was again mediated by upregulation of p27 and downregulation of CDK4 (Fig. 4C,D). Furthermore, similar to the miR-564 overexpression, MDA-MB-231 cells have lost their mesenchymal morphology in the presence of siCocktail observed by DAPI/Phalloidin staining (Fig. 4E). Using RTCA, we showed reduced ability of MDA-MB-231 cells to migrate and invade after siCocktail treatment (Fig. 4F). We concluded that miR-564 inhibitory effect on migration and invasion was, at least in part, *via* simultaneous targeting of AKT2, GNA12, GYS1 and SRF. Finally, we validated the association of miR-564 targets with cell invasiveness using 58 cancer cell lines in NCI60 panel and 12 breast cancer cell lines from GSE40059 dataset where higher levels of target genes expression in combination in invasive cell lines compared to less invasive cell lines were observed (Fig. 4G,H). However, individual target genes’ expression did not significantly correlate with invasiveness in both datasets (Supplementary Fig. 3A,B). Overall, we demonstrated that miR-564 inhibits proliferation, migration and invasion by targeting a network of genes found in PI3K and MAPK pathways.

### miR-564 expression predicts tumor progression and better survival in breast cancer patients

**Figure 5:**
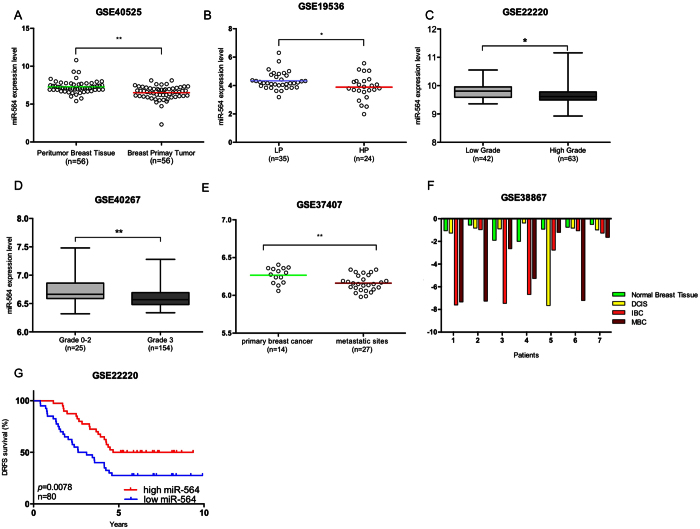
miR-564 predicts survival and suppresses tumor progression and metastasis in breast cancer patients. (**A**) miR-564 expression analysis in GSE40525 dataset containing 56 matched breast cancer patient samples. (**B**) miR-564 expression in breast cancer patients with low proliferative (LP) and high proliferative (HP) tumors in GSE19536 dataset. (**C,D**) miR-564 expression in breast cancer patients from GSE22220 and GSE40267 datasets grouped according to tumor grades. Data were used as it is provided. For GSE22220 dataset low and high grades were defined as grade 1 and 3, respectively. (**E**) Patient miR-564 levels in 14 primary and 27 metastatic tumors from GSE37407 dataset. (**F**) miR-564 expression levels in patients showing different stages in breast cancer tumor progression (GSE38867). (**G**) Distant Relapse Free Survival (DRFS) of breast cancer patients from GSE22220 dataset in correlation to miR-564 expression levels. Patients were separated from median miR-564 expression value and survival graph had a follow-up for 5 years.

The inhibitory effects of miR-564 on proliferation and migration of breast cancer cells led us to examine its association with clinic-pathological features of breast cancer patients. Importantly, analysis of GSE40525 dataset^39^ showed a lower miR-564 expression in breast primary tumors as compared to matched peritumor breast tissue suggesting that miR-564 could have a potential inhibitory effect on tumor formation (Fig. 5A).

In line with our data showing decreased EMT and migration upon miR-564 overexpression (Fig. 2), we have observed in breast cancer patient datasets that miR-564 expression negatively correlates with tumor aggressiveness and metastasis. A lower miR-564 expression was present in patients with high proliferative and high grade tumors as compared to patients with low proliferative and low grade tumors in GEO datasets with accession numbers GSE19536, GSE22220 and GSE40267^40^,^41^,^42^ (Fig. 5B–D). Furthermore, in a dataset having miRNA expression in primary tumors of 14 patients and their corresponding lymph nodes and distant metastasis samples (GSE37407), with unpaired analysis we found significantly lower levels of miR-564 in lymph nodes and distant metastatic sites as compared to primary tumors (Fig. 5E), which suggests a negative correlation of miR-564 with breast cancer metastasis. In line with all our previous findings relating miR-564 with suppression of tumor formation, progression and metastasis; in GSE38867 dataset having follow-up miRNA expression of 7 breast cancer patients at different stages of the disease, there was a gradual decrease in miR-564 levels as the disease progresses through a more aggressive stage (Fig. 5F). Strikingly, a higher miR-564 level is predictive of a significantly better distant relapse-free survival (DRFS) in breast cancer patients in GSE22220 dataset (Fig. 5G).

To further support the tumor suppressive roles of miR-564 in cancer, in general, we have analyzed several other datasets having miRNA expression of patients with different cancer types. Similar to our observations with breast cancer, miR-564 levels were lower in cancerous versus noncancerous tissues in a wide range of cancers including acute lymphoblastic leukemia (ALL), bladder cancer, osteosarcoma, prostate cancer, colon cancer and lung cancer (Supplementary Fig. 4A–D). In addition to this, more aggressive forms of pancreatic cancer and multiple myeloma were found to have lower miR-564 levels than their less aggressive counterparts (Supplementary Fig. 4E,F). These findings indicate that miR-564 is a potential tumor suppressor and may be utilized as a potential marker in cancer.

### Combined analyses of AKT2, GNA12, GYS1 and SRF expression mimic the effects of miR-564 on tumor progression and survival of breast cancer patients

**Figure 6:**
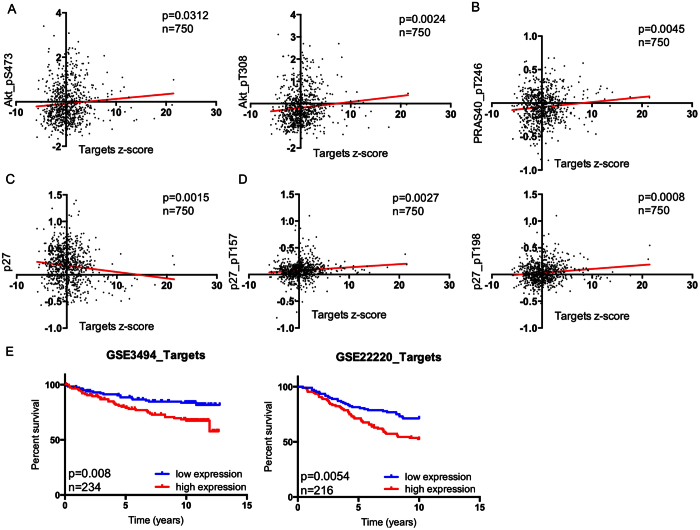
Expression of AKT2, GNA12, GYS1 and SRF in combination correlates with PI3K pathway activation in breast cancer patients and predicts survival. (**A**) Correlation analysis of AKT phosphorylations (S473 and T308) with combined mRNA expression levels of miR-564 target genes in 750 breast cancer samples from TCGA dataset. (**B**) PRAS40 phosphorylation (T246) and its correlation with mRNA levels of miR-564 target genes in 750 breast cancer samples from TCGA dataset. (**C**) Protein level correlation of p27 with mRNA levels of target genes in 750 breast cancer patients. (**D**) Correlation of p27 phosphorylation levels at two different sites (T157 and T198) with mRNA expression levels of targets in 750 breast cancer patient samples. (**E**) Survival analysis of breast cancer patients estimated by Kaplan Meier survival curve with high (red) and low (blue) expression of miR-564 target genes in combination using GSE3494 (n = 234) and GSE22220 (n = 216) datasets.

In order to further support the functional roles of miR-564 target genes in PI3K and MAPK pathways and G1/S transition by using breast cancer patient data, we have analyzed the correlation between mRNA expression of miR-564 targets and the expression/phosphorylation of PI3K and MAPK pathways and cell cycle proteins by using the Cancer Genome Atlas (TCGA) data^43^. In accordance with our own RPPA data showing negative regulation of Akt1 and Akt2 by miR-564, we observed that there is a positive correlation between the combinatorial expression of miR-564 targets and phosphorylation of Akt at both Thr308 and Ser473 residues (Fig. 6A,B). We also observed a positive correlation between the combinatorial expression of miR-564 targets and the phosphorylated form of an mTORC1 complex element, PRAS40 where Thr246 phosphorylation of PRAS40 by Akt causes its dissociation from mTORC1 complex leading to its activation^44^. Furthermore, mRNA expression of miR-564 targets was found to be negatively correlated with p27 protein and positively correlated with two different phosphorylated forms of p27: p27_pT157 and p27_pT198, both of which are mediated by Akt, and prevent p27 localization in nucleus and ultimately lead to cell cycle progression^45^. These results support the notion that miR-564 targets could be important regulators of PI3K pathway and G1/S transition in breast cancer patients.

Finally, using GSE3494 (n = 234) and GSE22220 (n = 216) breast cancer patient microarray datasets, we analyzed the effects of combinatorial expression of target genes on patient survival. When we combined the expression of all the targets with a z-score algorithm (details are given in Methods section), we found a better survival of patients with low expression of miR-564 target genes than patients with high miR-564 target genes expression (Fig. 6E,F). In both datasets, survival curves for individual miR-564 targets were also analyzed (Supplementary Fig. 5A,B). Results showed that combined analysis of all target genes, rather than that of the individual genes, predicts the survival of breast cancer patients more significantly. In conclusion, combined analyses of AKT2, GNA12, GYS1 and SRF expression mimic the effects of miR-564 on tumor progression and survival of breast cancer patients.

## Discussion

miR-564 has firstly been described together with six other miRNAs as potential blood-based biomarkers in schizophrenia patients^46^. Later report has identified downregulation of miR-564, miR-31 and miR-155 in chronic myeloid leukemia (CML) patients compared to non-CML patients. This study has been the first indicative of the role of miR-564 in cancer and also its association with MAPK, ErbB, mTOR and VEGF signaling pathways^47^. Recently, miR-564 has been recognized as a tumor suppressor miRNA in lung cancer by targeting ZIC3 protein^48^. However, the roles of miR-564 in breast cancer tumorigenesis and underlying mechanisms have not been reported yet. Our present study demonstrates a distinct regulatory role of miR-564 in breast cancer progression by direct targeting of a network of genes, namely AKT2, GNA12, GYS1 and SRF that leads to simultaneous inhibition of PI3K and MAPK pathways.

PI3K and MAPK pathways are among the top most commonly deregulated pathways in cancer. Effective interference with these pathways using appropriate inhibitors is of utmost importance in combating this disease. Currently, there are several available inhibitors exhibiting either selective or non-selective blockage of PI3K or MAPK pathways. Although satisfactory results were obtained in terms of initial response, many of these inhibitors were found to exhibit high toxicity, and rapid development of resistance was observed due to re-activation of the blocked pathway or activation of an alternative pro-survival pathway. Moreover, inhibition of either PI3K or MAPK pathway has been reported to trigger each other’s activity due to the presence of by-pass mechanisms in the form of positive or negative feedbacks as well as presence of proteins common to both pathways^13^,^49^,^50^. Several miRNAs have been demonstrated to target PI3K or MAPK pathways through different effectors and thus function to modulate tumor growth^29^,^51^,^52^,^53^,^54^,^55^,^56^. Xu *et al*. identified a pair of miRNAs inhibiting PI3K and MAPK pathways simultaneously by targeting two different closely related elements, IGF-IR and IRS1 at the upstream of these cascades^57^.

The four targets of miR-564 identified in the present study were proposed to act in concert to activate both PI3K and MAPK signaling pathways. Among these four miR-564 targets, AKT2, a serine-threonine kinase that contains SH2-like domain, is a well-known oncogene intensively studied in several cancers including breast cancer^58^,^59^. GNA12 is a guanine nucleotide-binding protein subunit and acts as a modulator of several signaling pathways. Its function has been validated in cardiac physiology^60^ and maintenance of blood pressure in smooth muscle^61^. GNA12 has also been reported as a mediator of invasion by upregulating IL-6 and IL-8 in MDA-MB-231 breast cancer cell line^62^. GYS1 is a member of glycogen synthase family and mainly overexpressed in muscle tissues and as the name refers, it functions in glycogen metabolism. Furthermore, AKT2 can activate GYS1 by phosphorylating and inhibiting glycogen synthase kinase 3B (GSK-3B) which in turn phosphorylates and inactivates GYS1^63^,^64^. SRF, serum response factor, functions as a regulator of c-fos and cell cycle progression as well as MAPK pathway downstream signaling, and it is a well-studied transcription factor in cancerous tissues^65^,^66^. Thus, the apparent link between these genes and proliferation or migration/invasion of cancer cells further supports the idea that miR-564 mediates its tumor suppressive effects, at least in part, through direct targeting and functionally inhibiting these four key genes.

PI3K pathway is known to induce EMT and invasion through several effectors, like Bmi1 and EZH2 which downregulate PTEN and thereby activate PI3K signaling^67^,^68^,^69^. Activated PI3K signaling can, in turn, upregulate inducers of EMT including Snail and Slug^70^ as well as Rho/Rac signaling through mTOR^71^. Similarly, activation of MAPK pathway by a mutant form of BRAF has also been shown to mediate migration and invasion by upregulating a RAS effector protein RhoA and promote acquisition of EMT phenotype^72^. In this study, we have shown the blockade of EMT and decreased migration and invasion capacity of breast cancer cells upon miR-564 overexpression, which suggests that this miRNA may also function as a metastasis suppressor by blocking these two pathways. Furthermore, our analysis on several cell lines and patient datasets has clearly supported the notion that a lower miR-564 expression may be associated with development of metastasis. Our analysis regarding the effect of target genes on survival of breast cancer patients revealed that similar to a better survival conferred by high levels of miR-564, patients expressing low levels of all four miR-564 targets exhibit a better survival in two different breast cancer datasets (Fig. 6A) further supporting the functional roles of these miR-564 targets in pathological conditions. Importantly, combinatorial expression analysis of four identified targets predict patient outcome better than their individual analysis. These findings further corroborate the necessity of blockade of a network of genes in order to obtain a desirable clinical outcome and supports potential use of miR-564 as a dual inhibitor of PI3K and MAPK pathways.

Collectively, our experimental results, corroborated with patient data, establish the miR-564/AKT2-GNA12-GYS1-SRF network as a key modulator of cell proliferation, EMT migration and invasion in breast cancer. Here, we report a potential tumor suppressor miRNA, miR-564, functioning not only in breast cancer cell lines, but also in breast and other cancer patients where miR-564 level was shown to be significantly downregulated in tumor tissues compared to adjacent normal tissues. Furthermore, miR-564 expression is also associated with the invasiveness of primary breast cancer tissue and metastasis. Importantly, higher levels of miR-564 expression in breast cancer patients lead to better survival. Finally, our study suggests that not individual, but only combinatorial knockdown of AKT2, GNA12, GYS1 and SRF mimics the functions of miR-564 as a simultaneous inhibitor of proliferation, EMT, migration and invasion of breast cancer supporting the notion that the mechanistic effects of miRNAs need to be examined at a network level to better elucidate their function.

## Methods

### RPPA data analysis and candidate miRNA selection

From our previously published RPPA data reporting the changes in the expression of 26 proteins in the presence of 733 miRNAs in MDA-MB-231 cell line^28^, we selected 16 key proteins that are components of PI3K and MAPK pathways and cell cycle. Proteins were classified into two groups as potential oncogenic and tumor suppressor ones according to their roles supported by literature. Each miRNA was correlated with these protein classifications, and cut-off for coefficient of variation was 0.25. Genefilter package^73^ was used for correlation (cut-off |0.5|) of miRNAs, and each miRNA was assessed as either a tumor suppressor miRNA or an oncomiR based on its regulatory effects on known oncogenes or tumor suppressors in the RPPA data (Supplementary Table 1).

### Cell culture

Human breast cancer cell lines (MDA-MB-231, MCF-7, BT-474 and MDA-MB-436) were obtained from ATCC (Manassas, VA, USA). MDA-MB-231 and MDA-MB-436 cells were cultured in Dulbecco Modified Eagle Medium (DMEM) (Lonza, NJ, USA) supplemented with 50 U/mL penicillin/streptomycin, 1% non-essential amino acids and 10% fetal bovine serum (Lonza, NJ, USA). The culturing media for MCF-7 and BT-474 cells were DMEM medium supplemented with 0.1% insulin, 50 U/mL penicillin/streptomycin, 1% non-essential amino acids and 10% fetal bovine serum. All cell lines were tested periodically for mycoplasma contamination using MycoAlert detection kit (Lonza, NJ, USA).

### Transfection with miRNA mimics, expression constructs and siRNAs

Transfections with siRNAs, miRNA mimics or hairpin inhibitors were carried out using Lipofectamine 2000 (Invitrogen) and OptiMEM medium (Gibco) as previously described^74^. miRNA mimics, siRNAs and hairpin inhibitors (Dharmacon, Lafayette, CO) were used in the final concentrations of 40 nM, 40 nM and 100 nM, respectively (for sequences see Supplementary Table 2). A combination of individual siRNAs targeting AKT2, GNA12, GYS1 and SRF was used as siCocktail (final concentration was 40 nM). The 3′-UTR vectors for AKT2, GNA12, GYS1 and SRF were transfected at 25 ng per well in 96-well plate format.

### *In vitro* viability, cell cycle, migration and invasion assays

End-point cell viability assays were performed by using either Cell Titer-Glo cell viability assay kit (Promega) or WST-1 reagent (Roche, Penzberg, Germany) as manufacturer’s protocol and were measured using Synergy HT microplate reader (BioTek, Vermont, USA). Real-time cell viability, migration and invasion assays were done on xCELLigence RTCA-DP (ACEA Biosciences Inc., CA, USA) according to manufacturer’s protocol. All RTCA measurements were normalized to transfection time, and statistical significance of the results was tested by paired two-tailed student t-test. Cell cycle analysis was performed using BrdU/7AAD flow kit (BD Biosciences, USA) as previously described^28^. For wound healing assay, 2.5 × 10^5^ cells per well were seeded in 24-well plates, and after 48 hours of incubation following the transfections and scratch formation, images were taken with 5x magnification using Nikon Eclipse inverted microscope (Nikon, Japan). All graphics and statistical analysis were carried out in GraphPad software (GraphPad software Inc., La Jolla, CA, USA).

### Lentiviral vector constructs and stable transfection

SMARTchoice human lentiviral hsa-miR-564 shMIMIC hCMV-turboRFP and GIPZ non-silencing lentiviral shRNA control were purchased from Dharmacon (Lafayette, CO). MDA-MB-231.luc cells (a kind gift from Dr. Dihua Yu (MD Anderson, TX)) were transduced with miR-564 viral particles in 24-wells plate, and stably transfected cells were selected with 1 μg/ml of puromycin 96 hours after transduction. To produce non-silencing shRNAs control containing viral particles, HEK293FT cells were co-transfected with 6 μg of GIPZ vector and 4.3 μl of trans-lentiviral packaging mix (Dharmacon) together with CaCl_2_ reagent (Dharmacon) in 6-wells plate. 48 hours post-transfection, first viral particles were harvested. Stably transfected cells were selected with 1 μg/ml of puromycin 96 hours after transduction.

### PolyHEMA assay

Anchorage-independent growth of cells was measured in 96-well plates pre-coated with polyHEMA. The images of cells were taken at 1, 3, 5 and 7 days after transfection. All images were taken at 5X magnification using Nikon Eclipse inverted microscope (Nikon, Japan). To quantify the proliferation of the same cells, WST-1 reagent (Roche, Penzberg, Germany) was added to each well and incubated for 4 hours. Following incubation, absorbance was measured at 450 nm using SynergyHT microplate reader (Biotek, VT, USA).

### DAPI/Phalloidin staining

Nuclear staining of MDA-MB-231 cells was done with 4,6-diamidino-2-phenylindole (DAPI) whereas F-actin was stained with Alexa Fluor 488 Phalloidin (1:40; Invitrogen) as previously described^75^. Slides were mounted (Shandon Immu-Mount, Thermo Scientific, Rockford, IL), and images were taken at 20x and 40x magnifications (Zeiss, Munich, Germany). Quantification of long axis/short axis ratio for each cell was performed as previously described^75^.

### 3′-UTR plasmid constructs, site directed mutagenesis and dual luciferase reporter assay

Predicted miR-564 binding sites at the 3′-UTRs of AKT2, GNA12, GYS1 and SRF were cloned at the downstream of luciferase reporter gene of psiCHECK™-2 Vector using *XhoI* and *NotI* restriction enzymes (for primer sequences see Supplementary Table 3). 3′UTR plasmid constructs were mutated in every other nucleotide of miR-564 seed sequence (in total 4 nucleotides, for primer sequences see Supplementary Table 3). MDA-MB-231 and MCF-7 were seeded in 96-wells plates and co-transfected with 40 nM of mimic or 100 nM hairpin inhibitors together with 25 ng of either 3′-UTR vectors or mutated constructs. After transfection of 48 hours, *firefly* and *renilla* activities were measured with dual luciferase reporter assay system (Promega). Luminescence reading was taken with Synergy HT microplate reader (BioTek, Vermont, US). *Renilla* luminescence intensities were normalized to internal *firefly* luminescence of psiCHECK-2 vector.

### Quantitative real-time PCR of miRNAs and mRNAs

Total RNA was isolated using TRIsure (Bioline, Luckenwalde, Germany) as manufacturer’s protocol. For cDNA synthesis, total RNA was reverse-transcribed using RevertAid RT Reverse Transcription kit (Life Technologies). Real-time PCR assay was performed using LightCycler^®^ 480 SYBR Green I Master kit (Roche) (for primer sequences see Supplementary Table 3), and ACTB and HPRT were used as housekeeping genes. Taqman microRNA reverse transcription kit and Taqman gene-specific microRNA assays (Applied Biosystems, Weiterstadt, Germany) were used for miRNA quantifications according to manufacturer’s protocol. RNU44 or RNU48 were used as housekeeping genes for miRNA qRT-PCRs. Both mRNA and miRNA data analyzed according to ΔΔ*C*_*T*_ method^76^.

### Western blot analysis

Whole-cell lysates were prepared with RIPA lysis buffer containing protease inhibitor complete Mini (Roche, Basel), anti-phosphatase PhosSTOP (Roche, Basel), 10 mM NaF and 1 mM Na_4_VO_3_ and quantified using BCA Protein Assay Reagent Kit (Thermo Scientific, Rockford, IL). 15 ug of total protein was loaded and separated by sodium dodecyl sulfate-polyacrylamide gel electrophoresis, transferred to PVDF membrane and probed with primary antibodies (Supplementary Table 4). Blots were developed using Amersham chemiluminescence kit (GE Health lifesciences, US).

### Bioinformatics analyses of target predictions, cell line and patient datasets

For the combinatorial target prediction analysis for miR-564, predicted targets in four different databases were downloaded. For TargetScan, only targets with conserved sites were selected. For DIANA and PITA, targets with a miTG score greater than 0.7 and a score of less than −10 respectively were included. No cut-off was made for predicted targets of miRWalk. Cell line and patient data were retrieved from NCI60 cell line panel and NCBI GEO databases (GSE40059, GSE40525, GSE19536, GSE22220, GSE40267, GSE37407, GSE38867, GSE31376, GSE40355, GSE28425, GSE14985, GSE29542, GSE37053 and GSE3494). Gene names were retrieved from specific platforms used in each dataset. In GSE40059, cell lines were reassigned as less invasive or invasive mostly according to their phenotype, invasive behavior and expression of the EMT marker genes^77^,^78^,^79^,^80^. SK-BR-3 and MDA-MB-468 cell lines were classified as undefined since they express low levels of E-cadherin although they are negative for two mesenchymal markers, Vimentin and Fibronectin, and also they have an epithelial-like or an intermediate morphology^77^,^78^,^80^. Therefore, they were excluded from both groups and indicated as undefined. For NCI60 cell lines, classification of less invasive and invasive was made by Park *et al*.^81^. TCGA mRNA and RPPA data were downloaded from cBioPortal for Cancer Genomics^82^,^83^ and TCPA databases^32^, respectively. Target-network expression was defined as sum of z-scores of individual genes. z-score of each gene is calculated as conventional z-score formula using mean and population standard deviation of a gene within patients^84^,^85^. In GSE40059 dataset, log2 expression values and the sum of z scores were normalized by subtracting the minimum value from each individual and dividing by its range. Two-tailed student t-test was used for comparisons between two patient groups with p = 0.05 significance cut-off. For survival graphs created by miR-564 expression, patients with less than 10 year distant-relapse free survival were selected 5 year cut-off was made on GSE22220 dataset. Survival graphs were generated by Kaplan-Meier method from Graphpad with removal of patients without any survival information or dead due to non-cancer cause. Patients were classified and separated from median of gene expressions. Log-rank test was used to calculate significance for survival plots.

## Additional Information

**How to cite this article**: Mutlu, M. *et al*. miR-564 acts as a dual inhibitor of PI3K and MAPK signaling networks and inhibits proliferation and invasion in breast cancer. *Sci. Rep*. **6**, 32541; doi: 10.1038/srep32541 (2016).

## Supplementary Material

Supplementary Information
