# Supplementary material for: miR-564 acts as a dual inhibitor of PI3K and MAPK signaling networks and inhibits proliferation and invasion in breast cancer
Source: Sci Rep. 2016 Sep 7;6:32541. doi: 10.1038/srep32541 (PMC5013276; doi:10.1038/srep32541)
Supplement: Supplementary Information [file srep32541-s1.pdf]

miR-564 acts as a dual inhibitor of PI3K and MAPK signaling networks and inhibits proliferation and invasion in breast cancer

Merve Mutlu[1#], Özge Saatci[1#], Suhail A. Ansari[1], Emre Yurdusev[1], Huma Shehwana[1], Özlen Konu[1], Umar Raza[1], Özgür Şahin[1*].

[1]Department of Molecular Biology and Genetics, Faculty of Science, Bilkent University, 06800 Ankara, Turkey

[#]These authors contributed equally to this work.

SUPPLEMENTARY INFORMATION

Supplementary Figures

Supplementary Figure 1. The effects of overexpression of two potential tumor suppressor miRNAs on cell viability.

Supplementary Figure 2. miR-564 target(s) selection based on dual inhibition of PI3K and MAPK pathways.

Supplementary Figure 3. Expression level of individual targets in cancer cell lines.

Supplementary Figure 4. Correlation of miR-564 expression with tumor progression and metastasis in patients with different types of cancers.

Supplementary Figure 5. Survival of breast cancer patients based on expression of individual miR-564 targets.

Supplementary Figure 6. Full-length blots of phospho-Akt (Thr308) and phospho-ERK1/2 proteins with low and high exposures for Figure 1C and 3D.

Supplementary Tables

Supplementary Table 1. List of potential tumor suppressor miRNAs selected according to their effects on PI3K and MAPK pathways as well as cell cycle proteins.

Supplementary Table 2. List of siRNAs, miRNA mimics and hairpin inhibitors.

Supplementary Table 3. List of primers used for qRT-PCR and cloning of 3’-UTRs.

Supplementary Table 4. List of antibodies used in Western blot experiments.

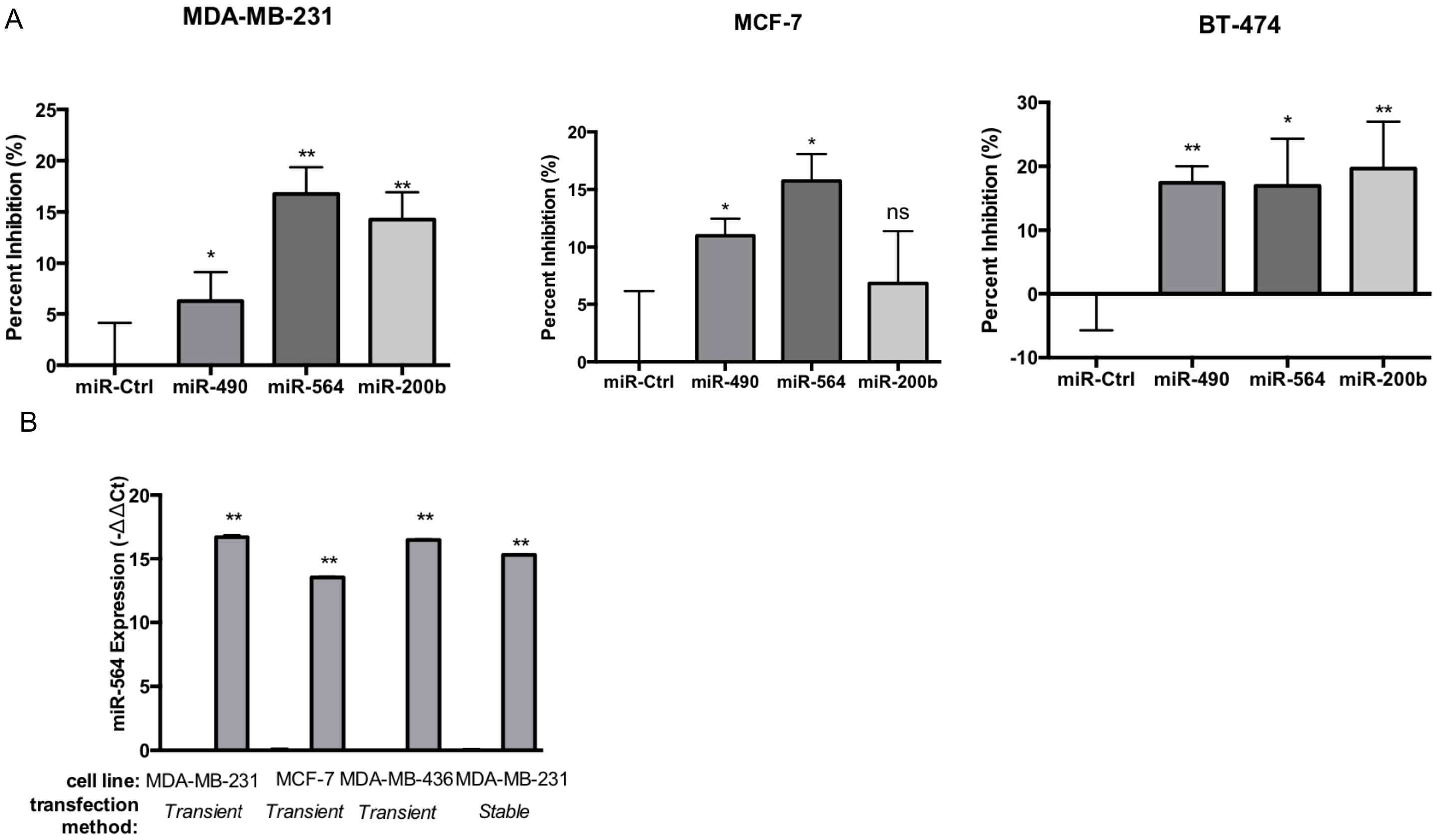

Supplementary Figure 1. The effects of overexpression of two potential tumor suppressor miRNAs on cell viability.(A) Cell viability assay was done upon overexpression of miR-564 or miR-490 in MDA-MB-231, MCF-7 and BT474 breast cancer cell lines. miR-200b transfected cells were used as a positive control. (B) Expression fold changes of ectopically expressed miR-564 in MDA-MB-231, MCF-7 and MDA-MB-436, together with miR-564 expression in stably transfected MDA-MB-231 cell line.

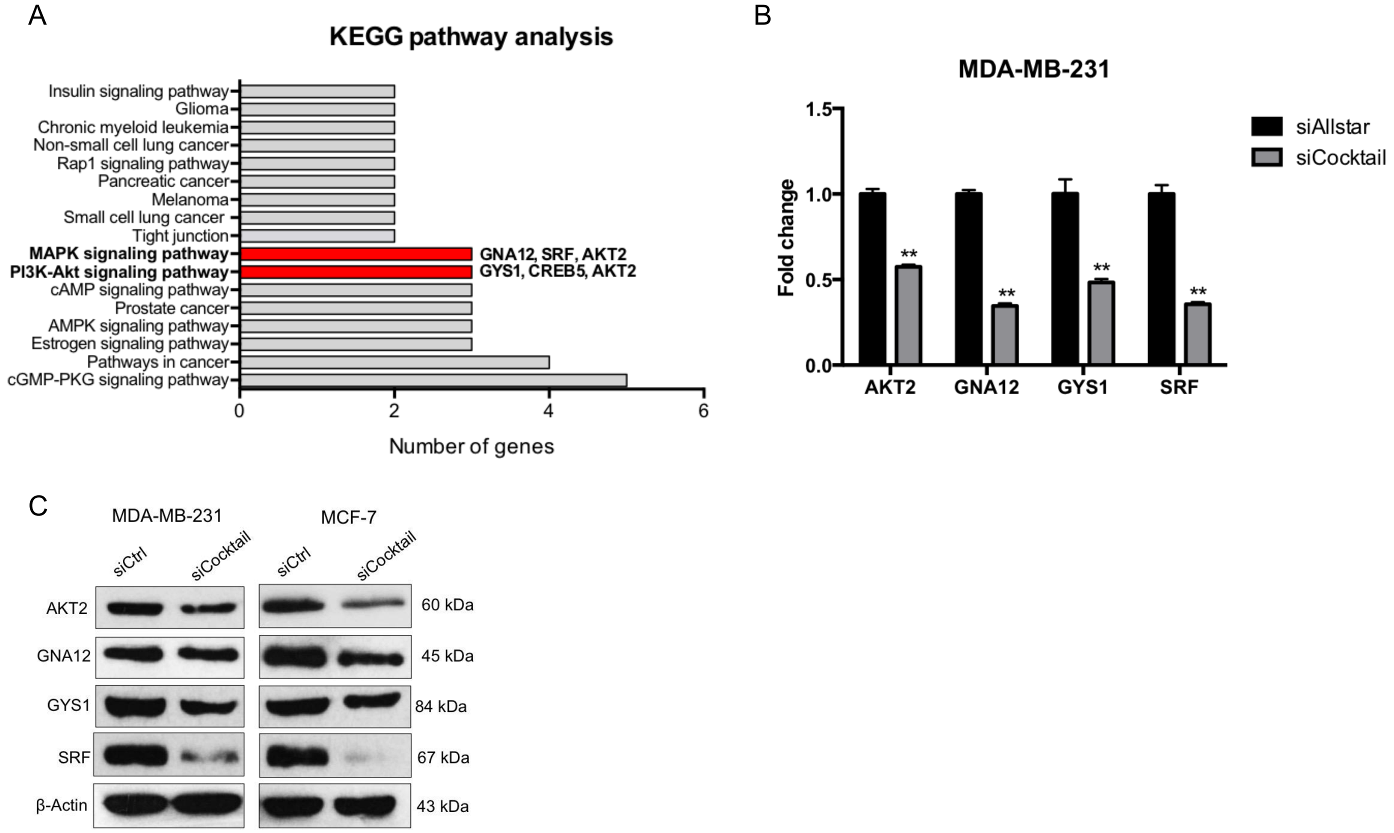

Supplementary Figure 2. miR-564 target(s) selection based on dual inhibiton of PI3K and MAPK pathways. (A) KEGG pathway analysis of miR-564 targets selected from 4 different target prediction algorithms. Genes that are linked with PI3K and MAPK pathways were selected for further experimental analysis. **(B)** qRT-PCR validation for downregulation of four target genes at mRNA level upon siCocktail transfections. **(C)** Western blot validation for downregulation of four target genes at protein level upon siCocktail transfection.

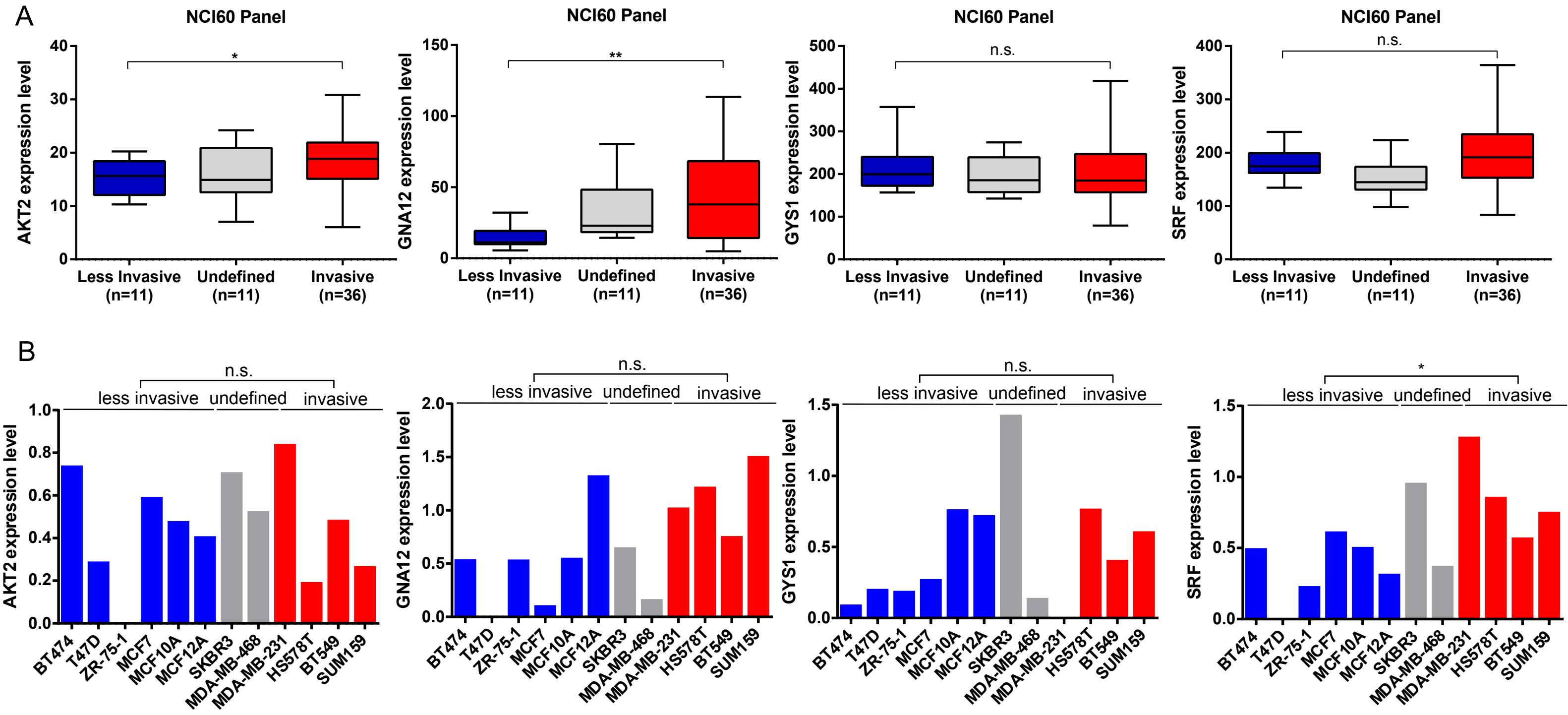

Supplementary Figure 3. Expression level of individual targets in cancer cell lines. (A) Analysis of expression of individual miR-564 targets in less invasive and invasive cells from NCI60 panel. **(B)** Expression of individual miR-564 targets in less invasive and invasive breast cancer cell lines panel as reported in GSE40059 dataset.

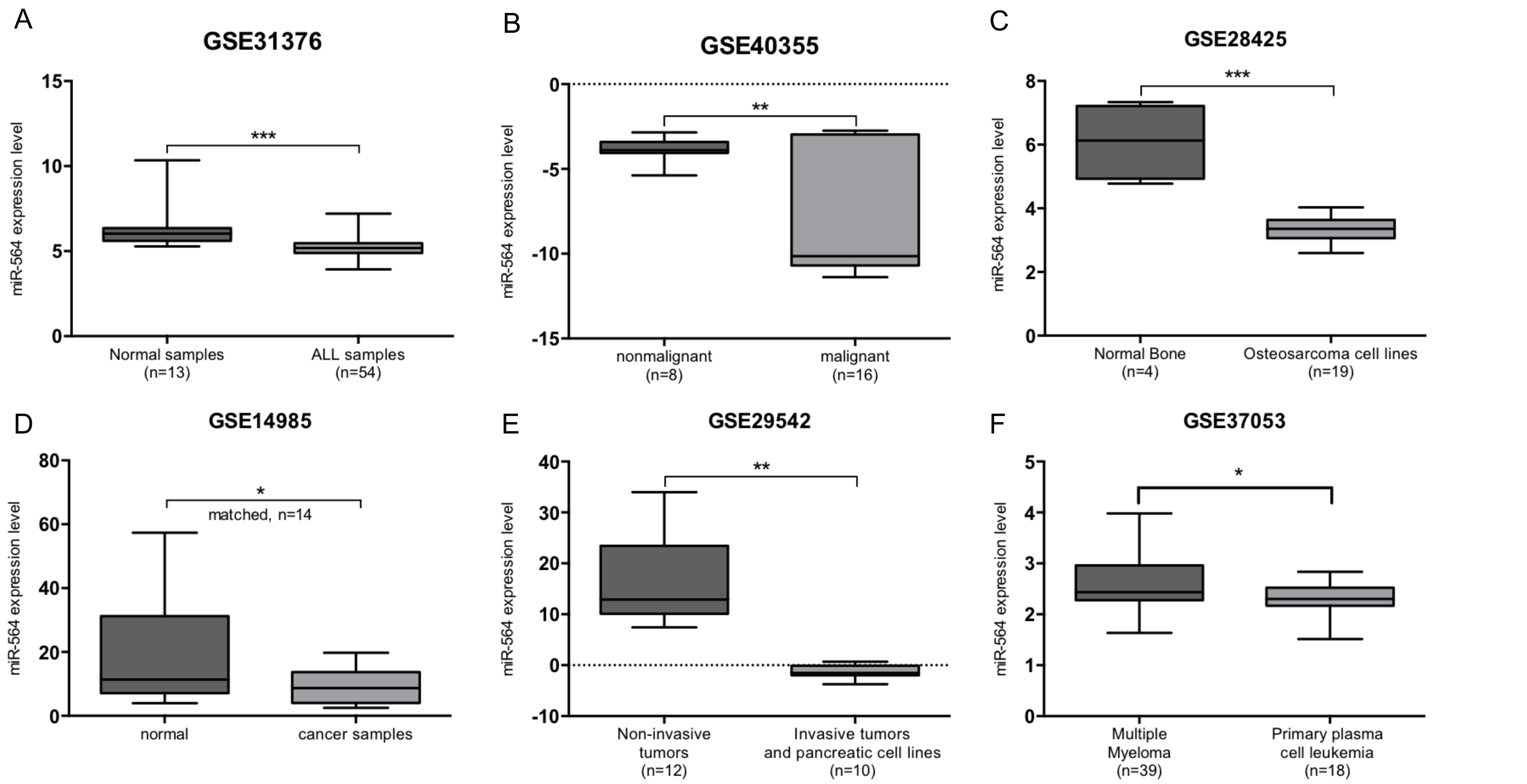

Supplementary Figure 4. Correlation of miR-564 expression with tumor progression and metastasis in patients with different types of cancers. (A) miR-564 expression levels in patients from GSE31376 dataset representing 13 normal and 54 ALL tumor samples. **(B)** miR-564 expression levels in non-malignant bladder cancer samples in patients from GSE40355 dataset. **(C)** miR-564 expression analysis in osteosarcoma cell lines compared to normal bone cell lines (GSE28425). **(D)** Analysis of miR-564 expression in 14 normal-tumor matched patient samples with lung, breast, prostate, colon, ovary cancer and lymphoma from GSE14985 dataset. Replicates in dataset were averaged. **(E)** Expression analysis of miR-564 level in bladder cancer types from GSE29542 dataset. **(F)** miR-564 expression levels in multiple myeloma and primary plasma cell leukemia from GSE37053 dataset.

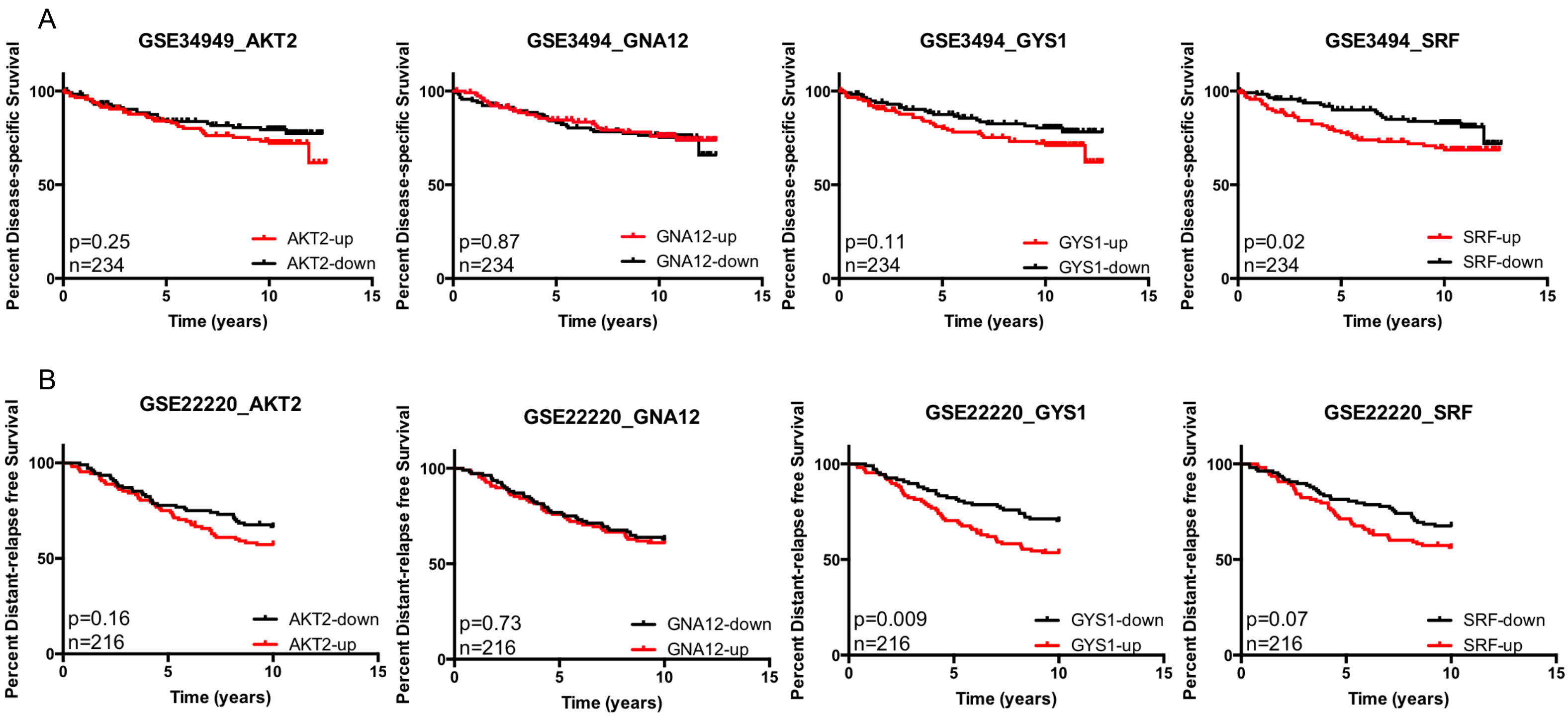

Supplementary Figure 5. Survival of breast cancer patients based on expression of individual miR-564 targets. (A) Survival analysis of breast cancer patients from GSE3494 dataset in relation to mRNA expression levels of miR-564 targets individually (n=234). **(B)** Graph showing survival correlations of breast cancer patients from GSE22220 dataset with mRNA level of individual miR-564 targets (n=216). Distant-relapse free survivals of patients were assessed with regard to high vs. low mRNA expressions.

A

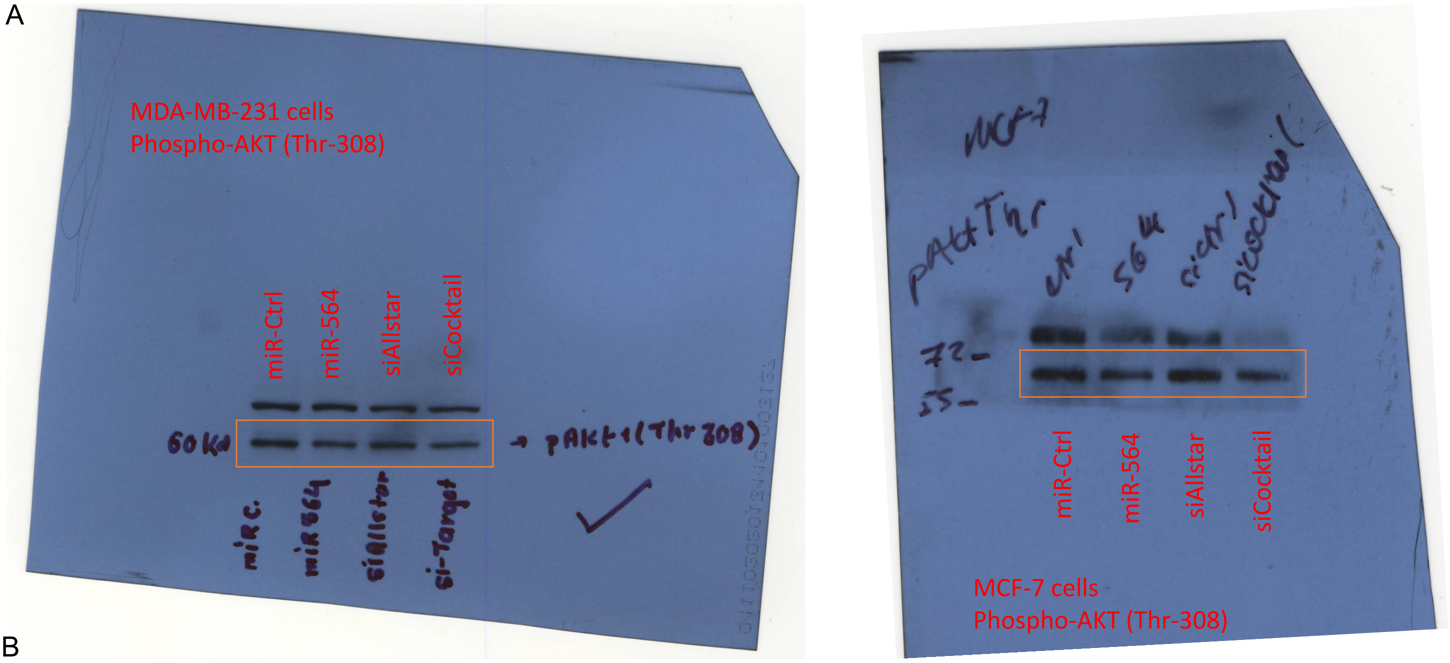

B

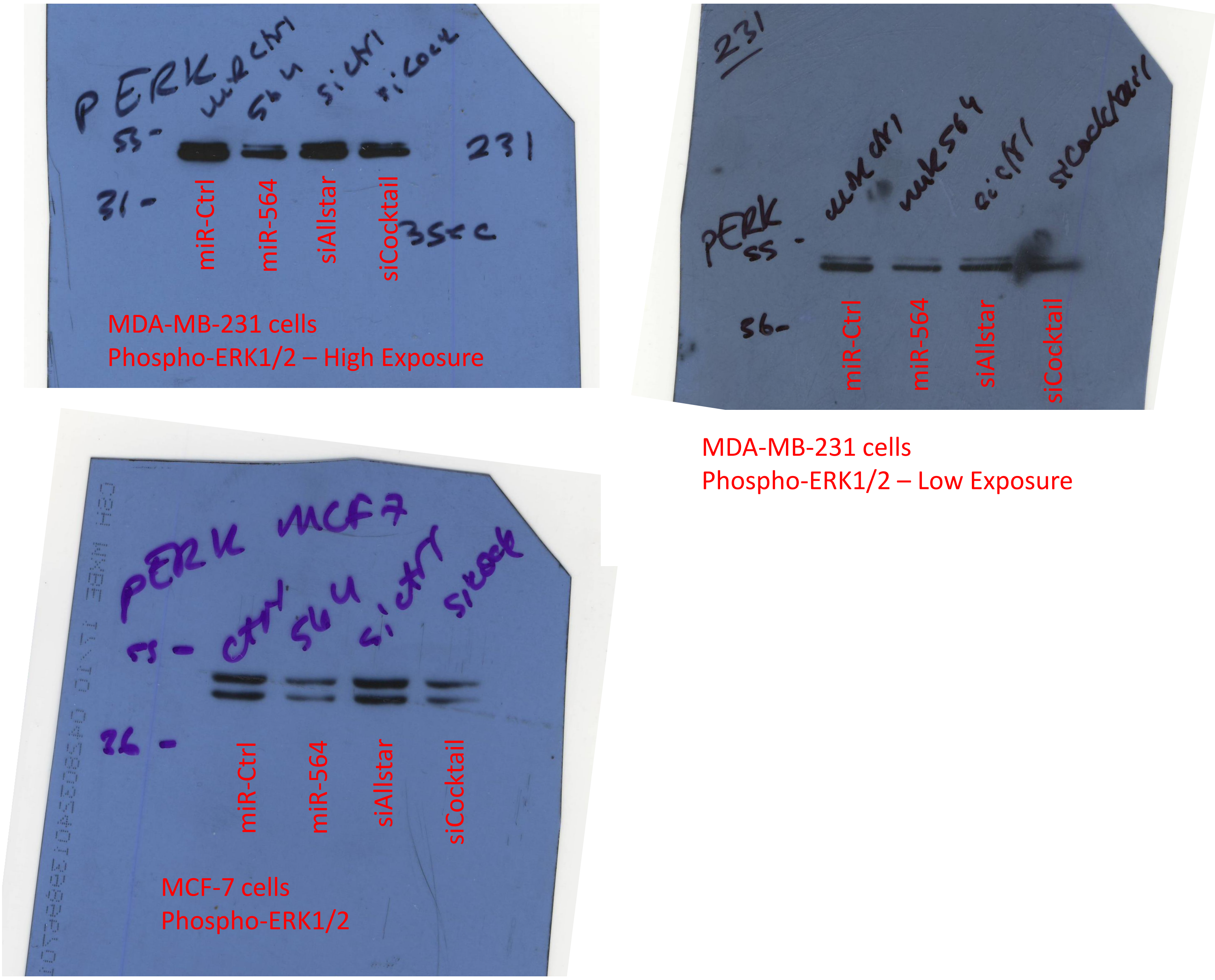

Supplementary Figure 6. Full-length blots of phospho-Akt (Thr308) and phospho-ERK1/2 proteins with low and high exposures for Figure 1C and 3D. (A) phospho-Akt (Thr308) western blots of MDA-MB-231 (left) and MCF-7 (right) cells transfected with miR-564 or siCocktail. **(B)** phospho-ERK1/2 western blots of MDA-MB-231 (top) and MCF-7 (bottom) cells transfected with miR-564 or siCocktail.

Supplementary Table 1. List of potential tumor suppressor miRNAs selected according to their effects on PI3K and MAPK pathways as well as cell cycle proteins.

Potential tumor suppressor miRNAs	Heatmap Correlation scores (cut off: -0.5)
hsa-miR-770-5p	-0.738
hsa-miR-323-5p	-0.688
hsa-miR-486-3p	-0.660
hsa-miR-96	-0.656
hsa-miR-206	-0.642
hsa-miR-886-3p	-0.623
hsa-miR-885-3p	-0.620
hsa-miR-223	-0.577
hsa-miR-634	-0.557
hsa-miR-182*	-0.545
hsa-miR-18a	-0.545
hsa-miR-129*	-0.541
hsa-miR-613	-0.539
hsa-miR-149*	-0.533
hsa-miR-512-3p	-0.529
hsa-miR-668	-0.522
**hsa-miR-564**	-0.520
hsa-miR-16	-0.517
hsa-miR-296	-0.516
hsa-miR-219-1-3p	-0.513
hsa-miR-452*	-0.513
hsa-miR-490	-0.505
hsa-miR-548b-5p	-0.502

Supplementary Table 2. List of siRNAs, miRNA mimics and hairpin inhibitors.

**siRNAs**	**Catalog number**	**Firm**
AKT2	D-003001-05	Dharmacon
AKT2	D-003001-21	Dharmacon
AKT2	D-003001-07	Dharmacon
AKT2	D-003001-08	Dharmacon
GNA12	D-008435-01	Dharmacon
GNA12	D-008435-02	Dharmacon
GNA12	D-008435-03	Dharmacon
GNA12	D-008435-04	Dharmacon
GYS1	D-017726-01	Dharmacon
GYS1	D-017726-02	Dharmacon
GYS1	D-017726-03	Dharmacon
GYS1	D-017726-04	Dharmacon
SRF	D-009800-01	Dharmacon
SRF	D-009800-03	Dharmacon
SRF	D-009800-05	Dharmacon
SRF	D-009800-06	Dharmacon
**miRNA mimics**		
miR-Ctrl	CN-001000-01-05	Dharmacon
miR-564	C-300882-01	Dharmacon
miR-490	C-300750-05	Dharmacon
miR-200b	C-300582-07	Dharmacon
**miRNA inhibitors**		
Negative Inhibitor	IN-001005-01-05	Dharmacon
miR-564 inhibitor	IH-300882-03	Dharmacon

Supplementary Table 3. List of primers used for RT-PCR and cloning of 3'UTRs.

**qPCR primers**	**Gene ID**	**Forward Primer**	**Reverse Primer**
CDH1	999	5'-CCCGGGACAACGTTTATTAC-3'	5'-GCTGGCTCAAGTCAAAGTCC-3'
ZO1	7082	5'-CAGAGCCTTCTGATCATTCCA-3'	5'-CATCTCTACTCCGGAGACTGC-3'
FN	2335	5'-CTGGCCGAAAATACATTGTAAA-3'	5'-CCACAGTCGGGTCAGGAG-3'
SNAI2	6591	5'-TGGTTGCTTCAAGGACACAT-3'	5'-GTTGCAGTGAGGGCAAGAA-3'
ZEB1	6935	5'-GGGAGGAGCAGTGAAAGAGA-3'	5'-TTTCTTGCCCTTCCTTTCTG-3'
ZEB2	9839	5'-AAGCCAGGGACAGATCAGC-3'	5'-CCACACTCTGTGCATTTGAACT-3'
AKT2	208	5'-CCATGAATGAGGTGTCTGTC-3'	5'-GGCCTCCAGGTCTTGATGTA-3'
GNA12	2768	5'-TCAAGAAGCACTTCCCGGACTTCA-3'	5'-TTTCACAGCATGGAACACGAAGCG-3'
GYS1	2997	5'-CCGCTATGAGTTCTCCAACAAGG-3'	5'-AGAAGGCAACCACTGTCTGCTC-3'
SRF	6722	5'-AGCACAGACCTCACGCAGA-3'	5'-GTTGTGGGCACGGATGAC-3'
CREB5	9586	5'-CGTGCCTCCTTGAAACAAGCCATT-3'	5'-ATGAAACACCAGCACCTGCCTAGA-3'
ACTB	60	5'-CCAACCGCGAGAAGATGA-3'	5'-CCAGAGGCGTACAGGGATAG-3'
HPRT	3251	5'-TGACCTTGATTTATTTTGCATACC-3'	5'-CGAGCAAGACGTTCAGTCCT-3'
**Cloning primers**			
AKT2_3'UTR		5'-GCGCGCCTCGAGATGACAGCCTGGGCTTACT-3'	5'-GCGCGCGCGGCCGCTACAGATGGATAGCTAGTTTATTACAG-3'
GNA12_3'UTR		5’-GGGTGTCTCGAGTCTGTGTGGTCCTTGAGTGG-3’	5’-GAAAGCGCGGCCGCAGGTAGCTGGACAAGTGAGC-3’
GYS1_3'UTR		5’-CTCCAGCCCTCGAGAGAGTGAGGACGAGGAGGAT-3’	5’-TCACACCCGCGGCCGCCTCCCCTCTCAACTGCAAAC-3’
SRF_3'UTR		5’-CGGGAGCTCGAGCAACCTCACCGAGCTACAGG-3’	5’-CTGGGCGCGGCCGCAATCTCCCCCACACACTGG-3’
AKT2_mut_3'UTR		5'-AAACCTGCCTGCCTCCCAGCCACTTTCATT ACTAGGGCTTCCTTCCAGCTGGCCTTACCT-3'	5'-AGGTAAGGCCAGCTGGAAGGAAGCCCTAGTAA TGAAAGTGGCTGGGAGGCAGGCAGGTTT-3'
GNA12_mut_3'UTR		5'-AAAGGGAGCACGTTGCCTTTTCAAACTCAC TTTCAGATTTCCTAAGAGCCCCTAGTCCAA-3'	5'-TTGGACTAGGGGCTCTTAGGAAATCTGA AAGTGAGTTTGAAAAGGCAACGTGCTCCCTTT-3'
GYS1_mut_3'UTR		5'-TTTCCAAGTTCCTGCACTCCAGAATCCACAA AGACTTTCATTTCTCTGGCTCCAGAATATGCATAAT-3'	5'-ATTATGCATATTCTGGAGCCAGAGAAATGAAA GTCTTTGTGGATTCTGGAGTGCAGGAACTTGGAAA-3'
SRF_mut_3'UTR		5'-ATGTTTGCCATGAGTATTAGCTTACCCAATGG GAACTTTCACCACCTCCCCACACACAGGCCTT-3'	5'-AAGGCCTGTGTGTGGGGAGGTGGTGAAAGTTC CCATTGGGTAAGCTAATACTCATGGCAAACAT-3'

Supplementary Table 4. List of antibodies used in western blot experiments.

Gene Name	Firm	Catalog Number	Dilution
phospho-AKT (Ser473)	Cell Signaling Technology	CST4058	1:1000
phospho-AKT (Thr308)	Cell Signaling Technology	CST4056	1:1000
total-AKT	Cell Signaling Technology	CST9272	1:1000
phospho-ERK1/2 (Thr202/Tyr204)	Cell Signaling Technology	CST4376	1:1000
ERK1/2	Cell Signaling Technology	CST4695	1:1000
p27/Kip1	BD Biosciences	610241	1:1000
p21	BD Biosciences	554228	1:1000
CDK4	Epitomics	2341-1	1:1000
CDK2	Sigma Aldrich	C5223	1:1000
phospho-Rb (Ser807/811)	Cell Signaling Technology	CST8516	1:1000
AKT2	Cell Signaling Technology	CST5239	1:1000
GNA12	Santa Cruz	sc-409	1:500
GYS1	Cell Signaling Technology	CST3886	1:500
SRF	Cell Signaling Technology	CST5147	1:500
E-cadherin	Cell Signaling Technology	CST5296	1:1000
ZO-1	Cell Signaling Technology	CST13663	1:1000
N-cadherin	Santa Cruz	sc-53488	1:1000
Vimentin	Cell Signaling Technology	CST5741	1:1000
Beta-actin	MP Biomedicals	69100	1:5000
